# Potentially Toxic Element Contamination of Dust from Bus Stops and Parking Lots in a Developing City, East China: Levels, Spatial Distribution, Source Analysis and Risk Evaluation

**DOI:** 10.3390/toxics14070593

**Published:** 2026-07-06

**Authors:** Ping Liu, Changqing Shan, Xingchao Qi, Shuo Li, Jidun Fang, Qiong Zhang, Kaipeng Zhang, Zaiwang Zhang

**Affiliations:** 1 Shandong Key Laboratory of Eco-Environmental Science for the Yellow River Delta, Shandong University of Aeronautics, Binzhou 256603, China; liuping@sdua.edu.cn (P.L.); zkpzh666@163.com (K.Z.); 2 Shandong Key Laboratory of Green Electricity & Hydrogen Science and Technology, Shandong Institute of Petroleum and Chemical Technology, Dongying 257061, China

**Keywords:** bus stop, parking lot, potentially toxic elements, source identification, risk assessment

## Abstract

Surface dust samples were collected from bus stops and parking lots in different functional areas of Binzhou City, Shandong Province, China. This study investigated the contamination characteristics, source apportionment, and potential ecological and health risks of potentially toxic elements (PTEs) in these dust samples. Eight target PTEs, including As, Zn, Pb, Cu, Cd, Cr, Ni, and Mn, were quantitatively analyzed. The results revealed distinct concentration differences in these elements between bus stop dust and parking lot dust. Several PTEs exceeded the corresponding local soil background values, predominantly Zn, Pb, Cu, Cd and Cr. Principal component analysis (PCA) indicated that Zn, Pb, Cu, Cr, Ni, and Mn in bus stop dust were mainly sourced from traffic emissions, whereas As and Cd primarily originated from atmospheric deposition. For parking lot dust, Zn, Pb, Cu, Cd, Cr, and Mn were predominantly attributed to traffic sources, while As and Ni were mainly derived from natural background sources. The geo-accumulation index (*I_geo_*) demonstrated that As, Cr, Ni, and Mn had negligible environmental impact, Pb, Cu, and Cd induced slight pollution, and Zn resulted in moderate pollution. Except for Cd, the average individual potential ecological risk index ($E_{r}^{i}$) values for all elements were below 40, suggesting a low ecological risk. Cd posed a moderate ecological hazard, whereas the comprehensive ecological risk index ($E_{r}^{i}$) values of all analyzed elements were at an extremely low level. The hazard index (HI) values via different exposure pathways and for all PTEs in both bus stops and parking lots were lower than 1, indicating no significant non-carcinogenic health risk. The carcinogenic risk ranking of elements was Cr > Cd > Ni > As, and their carcinogenic risk values (CR) via inhalation exposure were below 1 × 10^−6^, indicating no carcinogenic risk. This study provides a scientific basis for the environmental quality control and risk management of surface dust in urban bus stops and parking lots.

## 1. Introduction

Potentially toxic elements, as elements with properties like environmental persistence, high toxicity and bioaccumulation [1,2], are typical and priority-monitored pollutants which are ubiquitous in the environment and continuously migrate and transform [3,4], affecting both environmental quality [5,6] and human health [7,8]. Considering human health issues, metals such as As, Cd, Cr, and Ni, which are carcinogenic [9,10,11], have aroused widespread public and academic concern. Accordingly, systematic monitoring and risk assessment of PTE contamination is always of great necessity and significance.

In the urban environment, dust is an important carrier of pollutants, including PTEs, especially under a background of rapid industrialization and accelerated urbanization. Pollutants in dust can migrate to water bodies, soil, and the atmosphere through pathways like surface runoff and air movement, degrading urban environmental quality [11], and can also enter the human body through ways including dietary intake, inhalation, and dermal contact [12,13,14], directly or indirectly doing harm to public health. Traffic and transportation are among the primary contributors of urban population exposure to PTEs in dust, as commuters spend prolonged periods in traffic-intensive areas such as main roads, bus stops, subway entrances, and parking zones, where they are exposed to high-concentration dust pollution zones. PTE pollution in dust has been reported in urban roads [15], subways [16,17], and indoor surfaces [18,19].

Bus stops and parking lots are special functional areas in cities where large numbers of motor vehicles park daily, featuring intensive traffic flow and high population density and mobility. Studies have shown that vehicle exhaust [20], tires [21], and road construction materials [22] contain various PTEs that are released into the environment and subsequently accumulate in dust. Extensive investigations have been conducted on PTE pollution in urban road dust [23,24,25]. Nevertheless, targeted comparative research focusing on dust from bus stops and parking lots remains insufficient. The spatial differentiation of PTE enrichment, distinct source contributions and ecological health risks in these two typical traffic microenvironments have not been fully clarified, restricting targeted pollution prevention for urban public traffic facilities.

Binzhou, a typical developing industrial city located in the Yellow River Delta of East China, was selected as the research object in this work. A total of 58 surface dust samples were collected from 28 bus stops and 30 parking lots covering five urban functional zones. Eight target PTEs, including As, Zn, Pb, Cu, Cd, Cr, Ni, and Mn, were quantified via inductively coupled plasma–optical emission spectrometry (ICP-OES) detection. The three core research objectives are as follows: (1) to clarify the concentration levels, element composition and spatial differentiation characteristics of PTEs in dust from bus stops and parking lots; (2) to quantitatively distinguish natural and anthropogenic sources of PTE through Pearson’s correlation analysis and principal component analysis (PCA); and (3) to comprehensively evaluate the potential ecological risks and human non-carcinogenic/carcinogenic health risks via multiple assessment models. The findings of this study can supplement basic field data on traffic dust pollution in medium-sized developing cities in the Yellow River Delta, and provide scientific theoretical support for differentiated dust pollution control and risk management of urban bus stops and parking lots.

## 2. Materials and Methods

### 2.1. Study Area and Sampling

The study area was the central city area of Binzhou City in Shandong Province, China. Binzhou City is located in northern Shandong Province, China, covering a total area of 9660 km^2^ with a permanent population of 3.87 million. It lies within the Yellow River Delta, characterized by a warm temperate semi-humid continental monsoon climate with an annual average temperature of 14.8 °C and precipitation of 554.2 mm. As a developing industrial city, Binzhou achieved a GDP of 340.47 billion yuan in 2024, with a three-industry structure proportion of 9.2:45.5:45.3 (primary industry–secondary industry–tertiary industry) (www.binzhou.gov.cn). The urban core encompasses diverse functional areas including commercial zones, residential districts, educational institutions, and transportation hubs, providing representative settings for investigating urban dust contamination.

**Figure 1:**
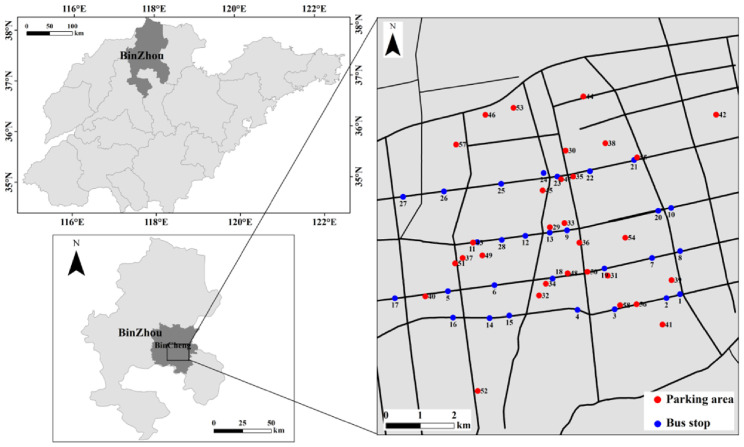
Distribution of sampling sites of the study area (Nos. 1–28: bus stop samples; Nos. 29–58: parking lot samples).

The distribution of sampling sites is shown in Figure 1. In general, 28 bus stops along four main arterial roads and 30 parking lots covering five types of functional zones which encompass a residential zone, a street-front commercial zone, a park, a hypermarket, and a hospital were selected as the sampling site. Dust samples were collected in December 2025. Winter witnesses the highest-level PTE pollution in atmospheric dust-fall across the study area throughout the whole year. These samples reflect metal enrichment features during peak-pollution periods. Though single-time winter sampling fails to show annual-average pollution characteristics, it reveals enrichment status and human-health risks under worst-case pollution conditions. During sampling, the ground remained dry without rainfall for more than 15 consecutive days. At each sampling site, surface dust within a certain surrounding scope was gently swept into polyethylene scoops with clean brushes, and no less than 300 g of dust sample was collected. A total of 58 surface dust samples were collected. Samples were brought back to the laboratory within the same day. In the laboratory, large-sized plant residues and gravel impurities were removed from the samples, and all dust samples were sieved using a 100-mesh nylon sieve with a pore size of 150 µm.

The particle size fraction below 100-mesh was adopted for subsequent testing in this study. Fine particles (<150 μm) are widely accepted and commonly used in most road dust-related studies, which is consistent with numerous published literatures, which facilitates result comparison with previous studies [26].

### 2.2. Potentially Toxic Element Determination

The digestion of the dust samples was performed using an aqua regia digestion method. In short, approximately 0.1000 g of sample was digested with 8 mL aqua regia mixed with guaranteed regent (GR) hydrochloric acid (HCl) and guaranteed reagent (GR) nitric acid (HNO_3_) at a volume ratio of 3:1 (HCl: HNO_3_ = 3:1, *v*/*v,* Sinopharm Chemical Reagent Co., Ltd., Shanghai, China) on an electric hot plate for 4 h. Following digestion and cooling, ultrapure water was added to reach a total volume of 50 mL for each sample. The digests were filtered and diluted to a constant fixed volume using ultrapure water. Elements including As, Zn, Pb, Cu, Cd, Cr, Ni, and Mn were analyzed by inductively coupled plasma–optical emission spectrometry (ICP-OES, iCAP PRO X Duo, Thermo Fisher, Bremen, Germany). The QA/QC procedures include 1 procedural blank, 3 parallel samples, and 1 certified reference material (ERM-S-510204, European Reference Materials, Geel, Belgium). The recovery rates of the targeted elements in the reference material ranged from 76.7% to 94.2%, and the standard deviations of elements in parallel samples were all lower than 5%.

The total digestion method utilizing hydrofluoric acid can extract PTEs trapped within silicate crystal lattices, chromium in particular. Nevertheless, such lattice-occluded PTEs are barely mobilized into the environment and thus contribute negligible environmental pollution. Accordingly, the aqua regia digestion method adopted in this study is rational for soil pollution assessment and human health risk evaluation. As a national standard pretreatment technique, this method complies with local soil-monitoring specifications, and the recovery rates of certified reference materials all satisfy the requirements of analytical quality control (Table S1).

### 2.3. Potential Toxic Elements Pollution Assessment

(1) Geo-accumulation Index Method

The geo-accumulation index [27] was chosen to evaluate the pollution degree of PTE in dust, calculated using Equation (1):

(1) $$I_{\mathrm{geo}}\;=\;\log_{2}(\frac{\mathrm{Ci}}{1.5\;\times \;\mathrm{Bi}})$$

where *I_geo_*, *C_i_*, and *Bi* stand for the geo-accumulation index, measured concentration of element *i* and reference value of element *i*, respectively. In the present study, soil background values of PTE of Binzhou City were chosen as the reference values for each element [28]. This background value originates from a province-wide soil survey of Shandong Province, which the team completed over more than 15 years, with 575 top-soil samples (0–20 cm) collected in Binzhou. As the team’s analytical protocols comply with local soil quality standards, this dataset serves as the regional background reference for the present study. Based on the values of *I_geo_*, the pollution degree of a single element in dust could be classified into seven degrees (Table 1).

(2) Potential Ecological Risk Index Method

The potential ecological risk index method was used to evaluate the pollution degree of PTE [29]. The potential ecological risk index for individual elements ($E_{r}^{i}$) was calculated using Equation (2):

(2) $$E_{r}^{i}\;={\;T}_{r}^{i}\;\times \;(C_{s}^{i}/C_{n}^{i})$$

where $E_{r}^{i}$, $C_{s}^{i}$, $C_{n}^{i}$, and $T_{r}^{i}$ denote the potential ecological risk index, measured concentration of PTEs *i*, background value of PTEs *i*, and toxicity coefficient of PTEs *i*, respectively. The toxicity coefficients for As, Zn, Pb, Cu, Cd, Cr, Ni, and Mn are 10, 1, 5, 5, 30, 2, 5, and 1, respectively [29].

The comprehensive potential ecological risk index (RI) equals the sum of $E_{r}^{i}$. The classifications of $E_{r}^{i}$ and RI are also shown in Table 2.

### 2.4. Human Health Risk Assessment

According to the revised health risk model of the US EPA, the human health risks from PTEs in dust were evaluated [26,30]. The daily exposure doses for non-carcinogenic risk PTEs were calculated as follows:

(3) $${\mathrm{ADD}}_{\mathrm{ing}}\;=\;\frac{C\;\times {IR}_{ing\;}\;\times \;\mathrm{EF}\;\times \;\mathrm{ED}\;\times \;\mathrm{CF}}{\mathrm{BW}\;\times \;\mathrm{AT}}$$

(4) $${\mathrm{ADD}}_{\mathrm{in}h}=\frac{C\;\times \;{\mathrm{IR}}_{\mathrm{in}h\;}\times \;\mathrm{EF}\;\times \;\mathrm{ED}}{\mathrm{BW}\;\times \;\mathrm{AT}\;\times \;\mathrm{PEF}}$$

(5) $${\mathrm{ADD}}_{\mathrm{der}}=\frac{C\;\times \;\mathrm{SA}\;\times \;\mathrm{SL}\;\times \;\mathrm{ABS}\;\times \;\mathrm{EF}\;\times \;\mathrm{ED}\;\times \;\mathrm{CF}}{\mathrm{BW}\;\times \;\mathrm{AT}}$$

where *ADDing*, *ADDinh*, *ADDder* are the average daily doses through ingestion, inhalation, and dermal contact (mg·kg^−1^·d^−1^), respectively. *C* is the metal concentration in dust (mg·kg^−1^). *IR_ing_* and *IR_inh_* are the ingestion rate of dust (mg·d^−1^) and inhalation rate of dust (m^3^·d^−1^). *EF*, *ED*, *BW*, *AT*, *PEF*, *SA*, *SL*, *ABS* and *CF* stand for the exposure frequency (d·a^−1^), exposure duration (a), average body weight (kg), averaging time (d), particle emission factor (m^3^·kg^−1^), exposed skin area (cm^2^), skin adherence factor (mg·cm^−2^·d^−1^), dermal absorption factor (dimensionless), and conversion factor (kg·mg^−1^), respectively.

For carcinogenic elements As, Cd, Cr, and Ni, the daily exposure dose through inhalation was calculated as follows:

(6) $${CADD}_{inh}=\frac{C\;\times \;{IR}_{inh}\;\times \;EF\;\times \;ED}{BW\;\times \;AT\;\times \;PEF}$$

where *CADD_inh_* and AT are chronic average daily dose through inhalation (mg·kg^−1^·d^−1^) and averaging time for carcinogens (d), and the other parameters are the same as mentioned above. The parameter values for the assessment model are shown in Table 3.

The health risk calculation formulas for PTEs dust are as follows:

(7) $${\mathrm{HQ}}_{i\;}=\;\frac{{\mathrm{ADD}}_{\mathrm{ij}}}{{\mathrm{RfD}}_{\mathrm{ij}}}$$

(8) $$HI=\sum HQ_{i}$$

(9) $$\mathrm{CR}\;=\sum R=\sum {\mathrm{CADD}}_{\mathrm{inh}}\times {\;SF}_{inh}$$

Here, *HQ_i_* is the non-carcinogenic hazard quotient for element *i*; *ADDij* is the average daily dose for metal *i* through exposure pathway *j* (mg·kg^−1^·d^−1^); *RfDij* is the reference dose for metal *i* through exposure pathway *j* (mg·kg^−1^·d^−1^).

*HI* is the total hazard index for non-carcinogenic risk; HI ≤ 1 indicates no non-carcinogenic risk, and HI > 1 indicates non-carcinogenic risk.

*R* stands for the carcinogenic risk for metal *i*; *SFinh* is the inhalation cancer slope factor for PTEs *i* (kg·d·mg^−1^) and *CR* is the comprehensive carcinogenic risk for multiple carcinogenic PTEs. CR < 1 × 10^−6^ indicates no carcinogenic risk, 1 × 10^−6^< CR < 1 × 10^−4^ indicates tolerable carcinogenic risk, and CR > 1 × 10^−4^ indicates intolerable carcinogenic risk. The reference values for *RfDij* and *SFinh* of targeted elements are shown in Table 4.

### 2.5. Data Analysis and Preprocessing

Statistical analyses in this study were mainly conducted with SPSS 20 software (SPSS Inc., Chicago, IL, USA), while Origin 2024 (OriginLab Corporation, Northampton, MA, USA) was utilized for data visualization and auxiliary data calculation. One-way analysis of variance (ANOVA) and Kriging interpolation were applied to reveal the spatial differentiation characteristics of eight potentially toxic elements across different functional zones. Principal component analysis (PCA) combined with Spearman’s correlation analysis and hierarchical cluster analysis (HCA) were performed to clarify the interrelationships, potential pollution sources and influencing factors of heavy metals.

## 3. Results and Discussion

### 3.1. Characteristics of Potentially Toxic Element Pollution

PTE concentrations in surface dust from bus stops and parking lots in Binzhou City are shown in Table 5. In bus stop dust, the concentrations (mean ± standard deviation) of As, Zn, Pb, Cu, Cd, Cr, Ni, and Mn were 4.43 ± 1.31, 284.71 ± 84.47, 54.21 ± 34.20, 57.70 ± 32.78, 0.39 ± 0.37, 77.57 ± 56.67, 26.88 ± 11.18, and 387.26 ± 87.87 mg/kg, respectively, with a descending order of Mn > Zn > Cr > Cu > Pb > Ni > As > Cd. In parking lot dust, the concentrations were 3.63 ± 1.27, 234.59 ± 103.38, 89.03 ± 86.52, 34.07 ± 19.55, 0.28 ± 0.21, 71.04 ± 32.03, 24.85 ± 12.09, and 463.71 ± 92.17 mg/kg, respectively, following the order of Mn > Zn > Pb > Cr > Cu > Ni > As > Cd. Compared with the soil background values of Binzhou City, the contents of As, Ni, and Mn in dusts from both bus stops and parking lots were lower than corresponding reference values, while those of Zn, Pb, Cu, Cd, and Cr exceeded the associated values. In bus stops, Zn, Pb, Cu, Cd, and Cr were 4.12, 2.45, 2.42, 2.65, and 1.13 times the background values, respectively, and those in parking lots were 3.39, 4.03, 1.43, 1.90, and 1.04 times the background values, respectively. This indicated that some metal elements were mainly from natural sources, while others might have external input problems.

Table 6 lists the PTE concentrations in road dusts from different regions. It could be observed that the levels of most elements in the present study were lower than those in other regions. Only a few elements showed relative higher levels. For example, Zn, Cu, and Cd were higher than in Chongqing [38], Pb was higher than in Beijing [36], Cr was higher than in Nanjing [39], Guangzhou [40], and Chongqing [38], Ni was slightly higher than in Beijing [36] and Guangzhou [40], and Mn was slightly higher than in Guangzhou [40]. The ranking order of metal concentrations in road dusts varied among different regions because of the complex effects of both natural conditions and anthropogenic factors.

**Figure 2:**
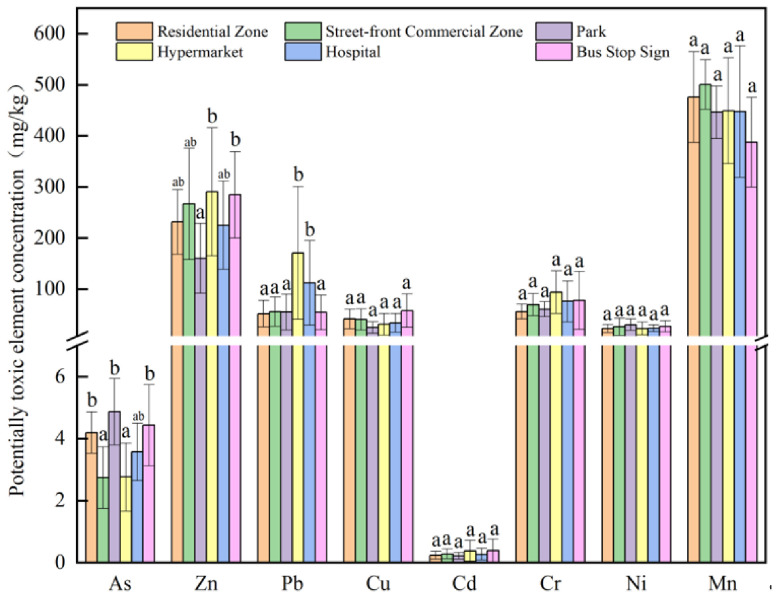
Potentially toxic element concentrations in dust from different functional areas (Note: Different lowercase letters (a, b, ab) indicate significant differences among samples at *p* < 0.05, whereas identical letters mean no significant difference between samples.).

The pollution levels of PTEs in dusts from parking lots in different functional areas (residential, street-front, park, hypermarket, and hospital) and bus stops are shown in Figure 2. Overall, Mn had the highest concentration, followed by Zn, and Cd had the lowest. The PTE concentrations decreased following different orders. In detail, in the parking lots, Mn > Zn > Cr > Pb > Cu > Ni > As > Cd, which was also observed for residential and street-front areas; Mn > Zn > Cr > Pb > Ni > Cu > As > Cd was observed for parks and Mn > Zn > Pb > Cr > Cu > Ni > As > Cd for hypermarkets and hospitals. The ANOVA results showed that As concentrations in residential areas, parks, and bus stops were significantly higher than in street-front areas and hypermarkets (*p* < 0.05); Zn concentrations in hypermarkets and bus stops were significantly higher than in parks (*p* < 0.05); Pb concentrations in hypermarkets and hospitals were significantly higher than in other functional areas (*p* < 0.05), while Cu, Cd, Cr, Ni, and Mn concentrations showed no significant differences among functional areas (*p* > 0.05).

Such PTE concentration discrepancies were closely associated with Binzhou’s industrial structure, traffic conditions, and urban development status. As a typical emerging industrial city in the Yellow River Delta, Binzhou was mainly dominated by light manufacturing, agricultural processing, and chemical supporting industries, without large-scale heavy metallurgy, non-ferrous smelting, and high-pollution automobile manufacturing industries, which caused serious PTE accumulation in megacities like Shanghai and Xi’an [41,42], and which fundamentally limited the urban dust PTE pollution level. In terms of traffic, Binzhou had far fewer vehicle holdings and road traffic volumes than first-tier and provincial capital cities [36,39]. Strict road dust and emission control policies reduced continuous traffic-related PTE emissions [43], whereas frequent vehicle braking and start–stop activities in residential and commercial areas triggered local enrichment of PTEs. In terms of urban construction, most sampling sites were newly built urban areas with complete road renovation and regular cleaning, which effectively reduced the accumulation of historical traffic pollutants compared with the old industrial urban areas [24,41]. Geologically, the Yellow River Delta was covered by alluvial loam with low PTE background values, leading to weak natural geological interference on surface dust pollution. Atmospheric transport of trace pollutants from surrounding chemical parks only formed local pollution hotspots instead of city-wide high PTE concentrations.

**Table 6:** Potentially toxic element concentrations in road dust from this study and other cities (mg/kg).

Cities/Country	As	Zn	Pb	Cu	Cd	Cr	Ni	Mn	References
Binzhou, China	4.03	259.65	71.62	45.89	0.34	74.31	25.87	425.49	This study
Yenimahalle, Ankara	-	97.98	55.22	3.81	52.45	66.88	38.37	-	[15]
Multan, Pakistan	18.91	257.35	53.43	49.46	-	90.15	38.6	38.19	[24]
Karachi, Pakistan	-	4254.40	426.60	332.90	62.30	148.10	389.70	-	[44]
Delhi, India		365.92	597.63	512.28	18.94	4816.94	-	-	[45]
Luanda, Angola	5	317	351	42	1.1	26	10	-	[46]
Narayanganj Sadar, Bangladesh	-	-	56.35	247.86	3.53	317.25	53.26	227.18	[23]
Dhaka, Bangladesh	8.09	239.16	18.99	49.68	11.64	144.34	37.01	-	[43]
Toronto, Canada	-	200.3	182.8	162	0.51	197.9	58.8	1202.2	[47]
Abeokuta, Nigeria	0.64	197.68	96.12	67.84	1.54	50.75	15.71	-	[25]
Kaifeng, China	8.66	287.07	172.67	76.25	0.74	126.48	27.68	-	[48]
Xiangtan, China	-	536.80	549.63	91.30	0.43	115.60	-	-	[49]
Beijing, China	-	-	50.79	63.73	0.47	77.36	23.60	564.12	[36]
Xian, China	78.67	279.00	119.73	50.66	8.82	138.35	30.99	-	[41]
Shanghai, China	-	687.25	212.94	186.41	0.97	218.91	64.91	-	[50]
Nanjing, China	-	302.7	82.65	102.83	4.37	67.13	46.24	-	[39]
Guangzhou, China	-	-	84.1	102	-	64.3	23.6	411	[40]
Chongqing, China	-	82.05	86.88	22.56	0.07	57.63	90.65	-	[38]

### 3.2. Spatial Distribution of Potentially Toxic Elements

**Figure 3:**
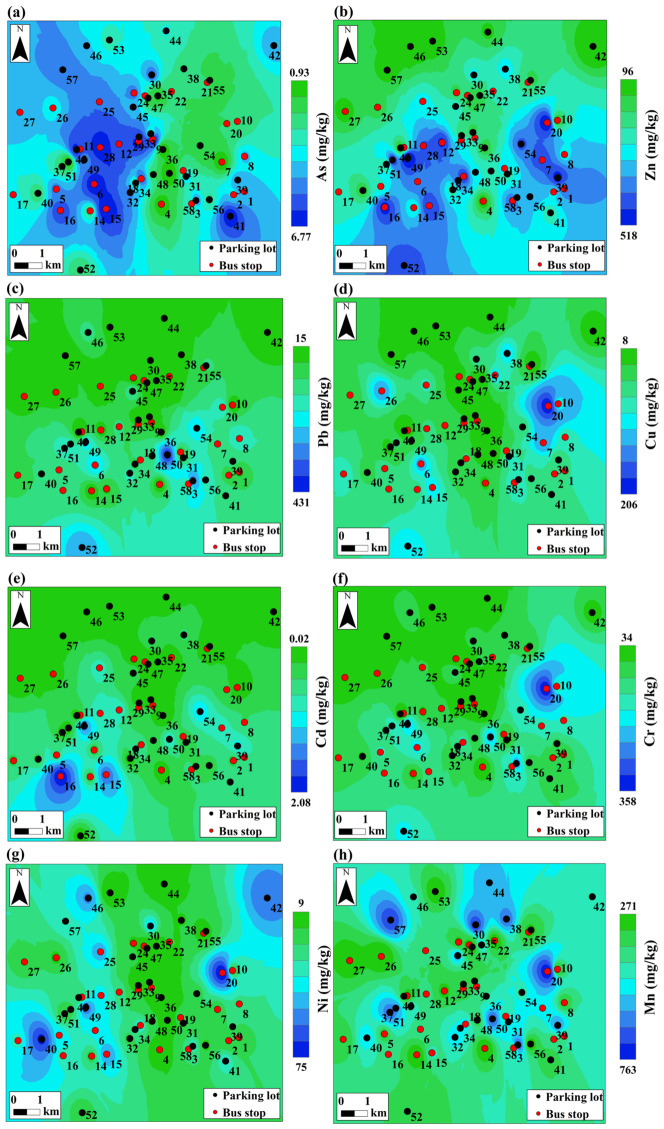
Concentration of potentially toxic elements in the dust samples at different locations of the city (Note: Nos. 1–28: bus stop samples (red dots); Nos. 29–58: parking lot samples (blue dots). (**a**) As (**b**) Zn (**c**) Pb (**d**) Cu (**e**) Cd (**f**) Cr (**g**) Ni (**h**) Mn.

The spatial distribution of eight potentially toxic elements in surface dust from bus stops and parking lots was mapped via Kriging interpolation (Figure 3). Except for As and Zn, the remaining elements exhibited obvious spatial differentiation characteristics of point-source pollution, and the high-value enrichment zones of different PTEs varied markedly. Overall, As, Zn, Ni and Mn were enriched at both functional zones (bus stops and parking lots) with widespread spatial distribution. The other four elements displayed distinct accumulation restricted to a single functional zone: Cu, Cd and Cr mainly accumulated at bus stops, while Pb was concentrated in parking lots and urban hypermarkets. Notably, Site 20 (a bus stop) presented synergistic high concentrations of Zn, Cu, Cr, Ni and Mn. Apart from contributions from typical traffic sources such as vehicle mechanical abrasion and exhaust emissions, this sampling site is adjacent to the Binbei Industrial Park. Dust deposition derived from industrial activities along the periphery constitutes a critical factor leading to multi-element co-enrichment and superimposed pollution at this location. As showed a typical area-source pollution pattern, with its hotspots mostly distributed at bus stops; seven high-As zones in the study area corresponded to bus stations, and only Parking Lot No. 41 was a local high-concentration point for As. High-value Zn sites occurred in both functional zones, including eight hotspots at bus stops and five at parking lots. Zn possessed a more scattered spatial distribution and relatively weak differentiation between the two functional zones.

Combined with the results of Q-mode hierarchical cluster analysis (HCA, Figure S1), the dust PTE pollution characteristics of bus stops and parking lots partially overlapped with similar enrichment rules of some elements, yet significant overall pollution differentiation existed between them. Such discrepancies mainly stemmed from differences in human activity patterns, vehicle flow conditions and supporting facilities at the two types of sites. At bus stops, frequent vehicle starting and stopping and dense pedestrian flow led to continuous inputs of abrasion debris from brake pads and tires, in addition to traffic exhaust, resulting in prominent accumulation of traffic-derived PTEs, including Cu, Cd and Cr. In contrast, vehicles stayed in idle or low-speed parking states for much longer in parking lots, and superimposed domestic pollution sources from surrounding parking lots contributed to higher Pb concentrations. Furthermore, spatial variations in industrial and commercial surroundings around the two functional zones further aggravated the divergent spatial distribution patterns of potentially toxic elements.

### 3.3. Source Identification of Potentially Toxic Elements

Potentially toxic elements in dust are influenced by both natural and anthropogenic factors, and similarities in their sources can lead to certain correlations between different elements [43,46]. Pearson’s correlation analysis results for PTEs in dust from bus stops and parking lots are shown in Table 7 and Table 8, respectively. It could be observed that in bus stops, As was not correlated with any other elements; Cd was only positively correlated with Zn; and all the other elements showed significant (*p* < 0.05) or extremely significant (*p* < 0.01) correlations from each other. This suggested that the sources of the PTEs varied and most elements had similar sources or environmental behaviors. In parking lots, As was only positively correlated to Ni; Ni also showed a significant correlation with Cd; Mn only showed a significant relationship with Cr; Cu showed significant relationships with Zn and Cr; other elements showed significant or extremely significant relationships. This indicated the sources of PTEs in dusts from those parking lots might be more complex.

**Figure 4:**
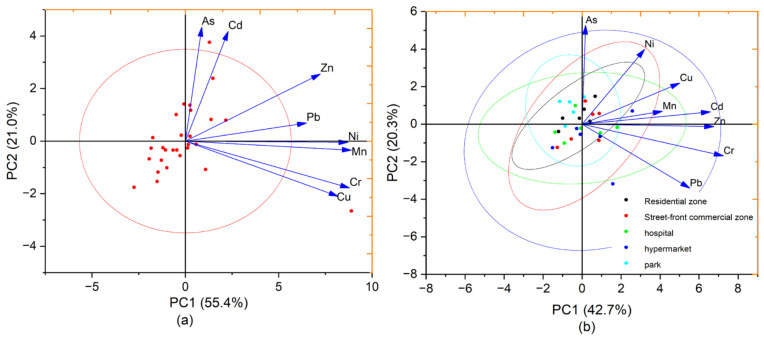
Principal component analysis (PCA) biplot showing the relationships among potentially toxic elements. (**a**) denotes bus stop surface dust, (**b**) denotes parking lot surface dust.

The results of principal component analysis (PCA, Figure 4 and Figure S2) and hierarchical cluster analysis (HCA, Figure S1) further clarified the co-occurrence patterns of potentially toxic element at bus stop and parking lot sampling sites. Samples from parking lots and bus stops differed slightly in spatial distribution, revealing distinct potential toxic elements pollution characteristics for the two site types. Bus stop samples featured a wider distribution range and higher dispersion, whereas parking lot samples showed tighter aggregation with more uniform pollution signatures.

Based on the loading characteristics of each element, samples collected from bus stops could be classified into four elemental assemblages (Figure 4a). Zn and Pb both showed positive loadings on Principal Component 1 (PC1) (Zn = 0.362, Pb = 0.325), as well as positive loadings on Principal Component 2 (PC2) (Zn = 0.358, Pb = 0.096). This indicated that the two elements shared consistent spatial distribution patterns and identical pollution sources. This group of elements was significantly enriched in densely populated areas, traffic corridors alongside administrative districts, urban parks, and parking lots of large supermarkets. They primarily represented urban non-point-source pollution derived from vehicle wear, superimposed with input from local industrial emissions. Ni and Mn exhibited moderate-to-high positive loadings on PC1 (Ni = 0.436, Mn = 0.445) and weak negative loadings on PC2 (Ni = −0.007, Mn = −0.049). These two elements were strongly correlated, and their spatially high-concentration zones were concentrated on the urban periphery, around hospitals, and near large downtown supermarkets with heavy pedestrian traffic. Their pollution sources were dominated by emissions from fossil fuel combustion for power generation and vehicle abrasion. Cu and Cr have high positive loadings on PC1 (Cu = 0.409, Cr = 0.440) and distinct negative loadings on PC2 (Cu = −0.294, Cr = −0.250). This pair of elements features the strongest intra-group correlation and clusters tightly in the PCA biplot, revealing highly overlapping pollution sources. Combined with their spatial distribution trends, this elemental assemblage was jointly controlled by vehicle emissions and minor industrial pollution inputs. Apart from natural sources, the six aforementioned elements mainly originated from traffic-related pollution sources, including vehicle exhaust emissions [20], tire wear debris [21], and road surface abrasion fragments [22]. As and Cd display unique differentiation characteristics. They carried extremely low loadings on PC1 (As = 0.044, Cd = 0.115) and the highest positive PC2 loadings among all detected elements (As = 0.607, Cd = 0.585). In the two-dimensional PCA space, they were fully separated from the other three elemental groups. As presented moderate enrichment along busy arterial roads near schools, administrative districts, and urban parks with dense human activity. The As concentrations within the study area were lower than the local soil background values, suggesting composite pollution sources: historical accumulated contamination formed by long-term atmospheric deposition [50,51], alongside newly added anthropogenic pollution released from vehicle exhaust, tires and road pavement materials [52,53].

For parking lots, two principal components were extracted, explaining 42.7% and 20.3% of the total variance, respectively. The factor loading distribution is shown in Figure 4b. Pb, Cu and Mn were classified as Group 1. All three elements presented positive loadings of 0.28–0.44 on Principal Component 1 (PC1) and positive loadings of 0.08–0.27 on Principal Component 2 (PC2), while their intra-group correlations were weak. Cr and Pb belong to Group 2, with positive loadings of 0.48 and 0.36 on PC1, and negative loadings of −0.29 and −0.25 on PC2, respectively. This group had the strongest intra-element correlations and clusters together in the PCA biplot, suggesting partial overlap of pollution sources. As and Ni formed Group 3. They showed low loadings of 0.01 and 0.21 on PC1 and the highest loadings of 0.66 and 0.50 on PC2, and were fully separated from the other three groups in the PCA space. Zn constituted an independent group, with a moderate positive loading of 0.45 on PC1 and a low negative loading of −0.02 on PC2. It was highly significantly correlated with Pb, Cu, Cd and Cr, indicating homologous pollution sources. The six aforementioned elements (Zn, Pb, Cu, Cd, Cr, Mn) were predominantly derived from traffic-related pollution sources. In contrast, As and Ni featured more complex source contributions, which were mainly attributed to the superposition of natural geogenic sources and vehicle emissions [53].

### 3.4. Potentially Toxic Element Pollution Assessment

**Figure 5:**
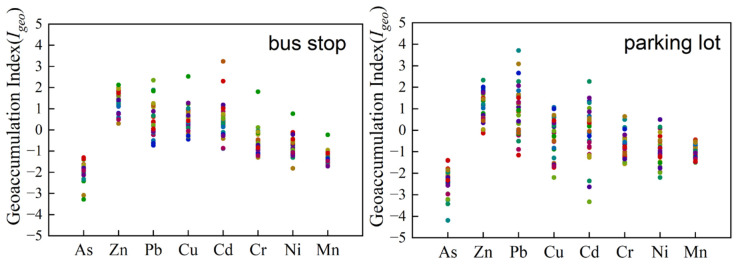
Geo-accumulation index of potentially toxic elements in dust.

The pollution degree and potential ecological risk of PTEs in dust were evaluated by calculating the *I_geo_* (Figure 5) and $E_{r}^{i}$ (Table 9). The *I_geo_* values for As, Zn, Pb, Cu, Cd, Cr, Ni, and Mn ranged from −4.19 to −1.31, −0.13 to 2.33, −1.16 to 3.71, −2.20 to 2.53, −3.33 to 3.24, −1.56 to 1.81, −2.19 to 0.76, and −1.72 to −0.23, respectively. The average *I_geo_* values (mean ± standard deviation) for As, Cr, Ni, and Mn were −2.16 ± 0.57, −0.61 ± 0.58, −0.90 ± 0.57, and −1.10 ± 0.31, respectively, all lower than 0, indicating negligible environmental impact of these four elements. The average *I_geo_* values for Pb, Cu, and Cd were 0.72 ± 1.01, 0.09 ± 0.91, and 0.19 ± 1.09, respectively, with mean values between 0 and 1, indicating slight pollution by these three PTEs. The *I_geo_* value for Zn was 1.21 ± 0.59, with a mean value greater than 1, indicating moderate pollution of Zn.

The results of *I_geo_* analysis were highly consistent with the spatial distribution characteristics of PTEs obtained via Kriging interpolation. Overall, Zn, Pb and Cd exhibited positive mean *I_geo_* values with obvious anthropogenic enrichment, which were strongly affected by human activities. Combined with the spatial differentiation map (Figure 3), high-Pb enrichment hotspots were predominantly distributed in parking lots. Site 50, which had the highest Pb concentration, was situated near large shopping malls, and vehicle-related wear and exhaust emissions served as the dominant source of Pb accumulation here. In comparison, Cd enrichment hotspots were clustered around bus stops; the peak Cd concentration appeared at Site 15 within the industrial park of the development zone. This zone hosted textile manufacturers including Weiqiao Textile and new material-processing enterprises, together with densely populated residential blocks. Accordingly, Cd accumulation in this region was driven by a combination of traffic pollutants and industrial atmospheric discharges. In contrast, As, Mn, Ni and Cr had negative average *I_geo_* values, and their concentrations were primarily controlled by natural geogenic sources with weak overall anthropogenic disturbance. Nevertheless, evident anthropogenic enrichment still occurred at partial sampling sites, indicating exogenous pollutant inputs could not be completely excluded. Distinct differences existed in the spatial variability in target elements. Pb and Cd showed the strongest variability, with standard deviations of 1.02 and 1.10 respectively. This demonstrated that the enrichment degrees of the two elements differed greatly across various urban functional zones such as bus stops and parking lots, presenting extremely high spatial heterogeneity.

As shown in Table 9, the $E_{r}^{i}$ values decreased following a general trend of Cd > Pb > Cu > Ni > Zn > As > Cr > Mn. Except for Cd, the average $E_{r}^{i}$ values for individual elements in dust samples were below 40, indicating low ecological risk from these PTEs. Cd posed a moderate ecological hazard. The average comprehensive ecological risk index (RI) was 108.05, below 150, indicating very low comprehensive ecological risk from multiple metal elements.

### 3.5. Human Health Risk Assessment of Potentially Toxic Elements

The average daily exposure doses (ADDs) and risk values (HI) for non-carcinogenic risk of PTEs in dust through ingestion, inhalation, and dermal contact for adult and child were calculated using the US EPA health risk model, with the results shown in Table 10a,b and Table 11a,b.

For adults, the HI values for different exposure pathways and all metal elements in both bus stops and parking lots were lower than the standard value of 1, indicating no non-carcinogenic health risk from the eight PTEs and three exposure pathways. This is similar to previous studies [54,55]. The HI value order for PTEs in bus stops was Cr > As > Pb > Mn > Cd > Cu > Ni > Zn, while the HI value for parking lot was Cr > Pb > As > Mn > Cd > Ni > Cu > Zn. The HI values for Pb and Mn in bus stop dust were lower than in parking lots, while other elements were higher in bus stops than in parking lots. For children, the HI value order for PTEs in both bus stops and parking lots was Cr > As > Pb > Mn > Cu > Ni > Zn > Cd. The HI values are consistently elevated in parking lots relative to bus stops, which implies higher PTE exposure risks for children within parking lot environments. This suggests that under current environmental conditions, exposure to PTEs in road dust via common pathways poses negligible non-carcinogenic risks to adults in these two functional areas. Despite the overall acceptable risk level, Cr, As and Pb still represent the dominant contributors to non-carcinogenic risk and should remain key targets for long-term environmental monitoring.

From the perspective of different exposure pathways, the total HI of eight PTEs differed noticeably between adults and children at both bus stops and parking lots. For adults, the ranking of risks across exposure pathways followed ingestion > inhalation > dermal contact, whereas for children, the ranking was ingestion > inhalation > dermal contact. For adults at bus stops, the HI value order for ingestion was Cr > Pb > As > Mn > Cu > Ni > Zn > Cd; for inhalation, it was Mn > Cr > As > Pb > Cu > Ni > Zn > Cd; and for dermal contact, it was Cr > As > Mn > Pb > Cd > Ni > Cu > Zn. For parking lots, the HI value order for ingestion was Pb > Cr > As > Mn > Ni > Cu > Zn > Cd; for inhalation, it was Mn > Cr > As > Pb > Ni > Cu > Zn > Cd; and for dermal contact, it was Cr > As > Mn > Pb > Cd > Ni > Zn > Cu. This result was slightly different from the conclusion reached by Zhang et al. that ingestion was the main exposure pathway in their study on road dust and green belt soils [54]. This indicates that dermal absorption poses the most significant non-carcinogenic health risk in roadside microenvironments, which should be prioritized in exposure prevention and control. This discrepancy might be attributed to regional environmental conditions, physicochemical properties of settled dust, and the US EPA exposure parameters adopted in this study. Binzhou is an emerging city that underwent urban expansion starting in 2003. Having developed for less than 30 years, the city features limited heavy industry, which results in low accumulation of potentially toxic elements in the environment. In addition, the parameter value of skin area adopted in this study was set to 5000 [36], which further lowered the risk contribution proportion of the ingestion pathway. The distinct ranking patterns of PTEs among ingestion, inhalation, and dermal pathways suggested that the health risks of different PTEs were highly pathway-dependent, and comprehensive risk management should be tailored accordingly.

**Figure 6:**
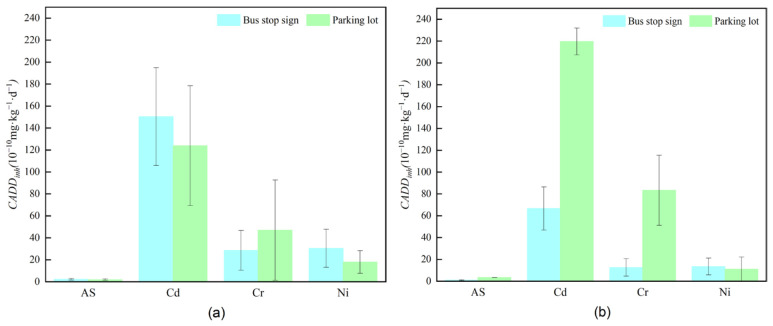
Daily respiratory exposure dose of carcinogenic potentially toxic element in dust (Panel (**a**): adult CADD; panel (**b**): child CADD).

The daily exposure doses and carcinogenic risk values for carcinogenic PTEs As, Cd, Cr, and Ni through inhalation are shown in Figure 6 and Table 12, respectively. The daily exposure dose order for PTEs for adults and children at both bus stops and parking lots was Cd > Cr > Ni > As, and the carcinogenic risks order was Cr > Cd > Ni > As. The average daily exposure doses and carcinogenic risks of As, Cd, and Ni for adults in bus stops were higher than those in parking lots, whereas Cr exhibited lower values at bus stops relative to parking lots. For children, the average daily exposure doses of Cd, Cr, and Ni in bus stops were higher than those in parking lots, while the carcinogenic risk of Cr and Ni in bus stops was higher than in parking lots. The comprehensive carcinogenic risk value (CR) for bus stops was lower than for parking lots, but both CR values lower less than 1 × 10^−6^, indicating inhalation exposure to PTEs poses no carcinogenic risk even though children exhibited higher risk values than adults. Previous studies had shown that the elevated lifetime carcinogenic risk in urban environments mainly originates from Cr, Ni, and As, which was consistent with the findings of this study [54,56]. Although Cr contributed the most to carcinogenic risk among the four metals, its individual risk remained within an acceptable range in both functional areas. The overall low carcinogenic risk suggests that inhalation exposure to these PTEs in roadside microenvironments did not pose an obvious cancer threat to the general population under current conditions.

In the present inhalation exposure evaluation, we used total dust concentrations rather than graded particle fractions. As reported in previous studies, particle size largely determines the penetration capacity of dust in the respiratory tract; inhalable and respirable particles are the main contributors to inhalation health risks. Considering that the inhalation hazard quotients of all detected elements were well below the critical value of 1.0, the inhalation health impact on the crowd was minimal. Accordingly, particle size classification was not conducted in our sampling and testing procedures. We acknowledge that this simplification may bring certain uncertainties to the assessment results.

## 4. Limitations

Several limitations of this study should be noted. First, all dust samples were collected at a single time point via one-off sampling, representing static instantaneous observations. Therefore, this work failed to reflect the seasonal dynamic variations in potentially toxic elements throughout the year in Binzhou City, and the pollution levels might be overestimated. Second, aqua regia digestion was adopted in the experiment, which only yielded pseudo-total element concentrations and imposed limitations on the subsequent ecological and human health risk assessments. Furthermore, although spatial analysis and statistical methods were applied to improve the reliability of pollution-source identification, this study lacked in-depth source apportionment based on the positive matrix factorization (PMF) model. In addition, the health risks of different exposed populations were not analyzed in detail. Future research can implement year-round seasonal sampling and adopt complete acid digestion to accurately quantify the contribution rates of various pollution sources, as well as systematically interpret risk characteristics across diverse populations and exposure pathways.

## 5. Conclusions

Bus stops and parking lots are functional areas characterized by heavy vehicle traffic and population mobility. PTEs in dusts from those zones in a developing city were determined. In general, the concentrations of As, Zn, Pb, Cu, Cd, Cr, Ni, and Mn in dust from bus stops and parking lots in Binzhou City were generally at low-to-moderate levels. Zn, Pb, Cu, Cd, and Cr were observed with concentrations above local soil background values. The sources of PTEs varied. As regards those in bus stops, traffic sources including vehicle exhaust emissions, tire wear, and road surface materials, and atmospheric deposition may represent major sources, while PTEs in parking lot dust might mainly derive from traffic sources and natural sources. The geo-accumulation index indicated that As, Cr, Ni, and Mn had negligible environmental impact, Pb, Cu, and Cd caused slight pollution, and Zn caused moderate pollution. Cd posed a moderate ecological hazard, while other elements showed low ecological risk, and the comprehensive ecological risk was very low. PTEs in dust from bus stops and parking lots showed no significant non-carcinogenic or carcinogenic health risks. This study suggests implementing routine monitoring of potentially toxic elements in traffic dust, alongside enhanced dust cleaning and environmental control measures for roads, bus stops, parking lots and other public areas, with special focus on the monitoring and control of Cd. Our results provided basic information for understanding dust as a pollutant carrier in traffic–residence zones in developing cities.

## Figures and Tables

**Table 1:** Classification standards of pollution degree based on geo-accumulation index.

Class	I	II	III	IV	V	VI	VII
*I*_geo_	≤0	0–1	1–2	2–3	3–4	4–5	>5
Pollution Degree	No pollution	Slight pollution	Moderate pollution	Moderate severe pollution	Severe pollution	Relatively severe pollution	Extremely severe pollution

**Table 2:** Classification standards of pollution degree based on potential ecological risk index.

Class	$E_{r}^{i}$	Hazard Degree	Class	RI	Hazard Degree
I	≤40	Slight	I	≤150	Slight
II	40–80	Moderate	II	150–300	Moderate
III	80–160	Strong	III	300–600	Strong
IV	160–320	Very strong	IV	>600	Extremely strong
V	>320	Extremely strong			

**Table 3:** Human health risk exposure parameter value.

Parameter	Unit	Adult Parameter Value	Child Parameter Value	Reference
C	mg·kg^−1^	Measured value	Measured value	This study
IR_ing_	mg·d^−1^	100	200	[31]
IR_inh_	m^3^·d^−1^	12.8	8.6	[32,33]
EF	d·a^−1^	350	320	[34]
ED	a	24	6	[31]
BW	kg	58.6	20.3	[33,35]
AT	cm^2^	ED × 365 (non-carcinogenic), 70 × 365 (carcinogenic)		[34]
PEF	m^3^·kg^−1^	1.36 × 10^9^		[31]
SA	cm^2^	5000	1150	[32,36]
SL	mg·cm^−2^·d^−1^	1	1	[36]
ABS	Dimensionless	0.03 (As), 0.001 (other elements)		[31]
CF	kg·mg^−1^	1.00 × 10^−6^		[34]

**Table 4:** Non-carcinogenic risk reference dose values and carcinogenic risk slope reference values.

Element	RfD_ij_/(mg·kg^−1^·d^−1^)			SF_inh_/(kg·d·mg^−1^)
	Ingestion	Inhalation	Dermal	Inhalation
As	3.00 × 10^−4^	1.23 × 10^−4^	3.00 × 10^−4^	4.30 × 10^−3^
Zn	3.00 × 10^−1^	3.00 × 10^−1^	6.00 × 10^−2^	-
Pb	3.50 × 10^−3^	3.52 × 10^−3^	5.25 × 10^−4^	-
Cu	4.00 × 10^−2^	4.02 × 10^−2^	1.20 × 10^−2^	-
Cd	1.00 × 10^−3^	1.00 × 10^−3^	1.00 × 10^−5^	6.30
Cr	3.00 × 10^−3^	2.86 × 10^−5^	6.00 × 10^−5^	42.00
Ni	2.00 × 10^−2^	2.06 × 10^−2^	5.40 × 10^−3^	0.84
Mn	4.70 × 10^−2^	1.40 × 10^−5^	2.40 × 10^−3^	

Note: Mn from reference [36]; other elements from reference [37].

**Table 5:** Potentially toxic element concentrations in surface dust from bus stops and parking lots (mg/kg).

Potentially Toxic Elements		As	Zn	Pb	Cu	Cd	Cr	Ni	Mn
Bus Stop	Maximum	6.78	452.83	168.44	206.16	2.08	358.64	75.03	763.49
	Minimum	1.72	127.86	19.95	26.15	0.12	41.60	12.59	271.84
	Mean	4.43	284.71	54.21	57.70	0.39	77.57	26.88	387.26
	SD	1.31	84.47	34.20	32.78	0.37	56.67	11.18	87.87
	CV	0.30	0.30	0.63	0.57	0.95	0.73	0.42	0.23
Parking Lot	Maximum	6.40	520.94	432.82	74.83	1.06	159.59	62.33	657.14
	Minimum	0.92	94.60	14.83	7.79	0.02	34.73	9.68	317.14
	Mean	3.63	234.59	89.03	34.07	0.28	71.04	24.85	463.71
	SD	1.27	103.38	86.52	19.55	0.21	32.03	12.09	92.17
	CV	0.35	0.44	0.97	0.57	0.77	0.45	0.49	0.20
Background value		11.2	69.1	22.1	23.8	0.147	68.4	29.5	597

**Table 7:** Pearson’s correlation coefficients of potentially toxic elements in bus stop dust.

	As	Zn	Pb	Cu	Cd	Cr	Ni	Mn
As	1.000							
Zn	0.308	1.000						
Pb	0.168	0.550 **	1.000					
Cu	−0.206	0.455 *	0.490 **	1.000				
Cd	0.330	0.519 **	0.143	−0.027	1.000			
Cr	−0.150	0.514 **	0.578 **	0.909 **	−0.005	1.000		
Ni	0.188	0.611 **	0.480 **	0.793 **	0.185	0.867 **	1.000	
Mn	0.032	0.702 **	0.512 **	0.782 **	0.176	0.886 **	0.862 **	1.000

Note: * indicates significant correlation at *p* < 0.05 level; ** indicates extremely significant correlation at *p* < 0.01 level.

**Table 8:** Pearson’s correlation coefficients of potentially toxic element in parking lot dust.

	As	Zn	Pb	Cu	Cd	Cr	Ni	Mn
As	1.000							
Zn	−0.05	1.000						
Pb	−0.282	0.386 *	1.000					
Cu	0.228	0.605 **	0.174	1.000				
Cd	0.098	0.763 **	0.405 *	0.328	1.000			
Cr	−0.135	0.651 **	0.838 **	0.397 *	0.608 **	1.000		
Ni	0.364 *	0.154	−0.004	0.276	0.376 *	0.263	1.000	
Mn	0.084	0.226	0.282	0.268	0.291	0.401 *	0.218	1.000

Note: * indicates significant correlation at *p* < 0.05 level; ** indicates extremely significant correlation at *p* < 0.01 level.

**Table 9:** Potential ecological hazard index of a single element and comprehensive potential ecological hazard index of potentially toxic elements.

		$E_{r}^{i}$								RI
		As	Zn	Pb	Cu	Cd	Cr	Ni	Mn	
Bus stop	Maximum	6.05	6.55	38.11	43.31	424.85	10.49	12.72	1.28	469.90
	Minimum	1.54	1.85	4.51	5.49	24.40	1.22	2.13	0.46	51.15
	Mean	3.96	4.12	12.26	12.12	79.79	2.27	4.56	0.65	119.73
	SD	1.17	1.22	7.74	6.89	75.43	1.66	1.89	0.15	79.44
	Ecological hazard	Slight	Slight	Slight	Slight	Moderate	Slight	Slight	Slight	Slight
Parking dot	Maximum	5.71	7.54	97.92	15.72	216.79	4.67	10.57	1.10	286.34
	Minimum	0.82	1.37	3.36	1.64	4.48	1.02	1.64	0.53	22.46
	Mean	3.24	3.39	20.14	7.16	56.14	2.08	4.21	0.78	97.14
	SD	1.13	1.50	19.57	4.11	43.02	0.94	2.05	0.15	58.19
	Ecological hazard	Slight	Slight	Slight	Slight	Moderate	Slight	Slight	Slight	Slight

**Table 10:** (**a**) Average daily exposure doses of potentially toxic elements in dust through different exposure pathways for adults (mean values). (**b**) Average daily exposure doses of potentially toxic elements in dust through different exposure pathways for children (mean values).

Potentially Toxic Element	Bus Stop			Parking Lot		
	ADD_ing_	ADD_inh_	ADD_der_	ADD_ing_	ADD_inh_	ADD_der_
(**a**)						
As	7.25 × 10^−6^	6.83 × 10^−10^	1.09 × 10^−5^	5.93 × 10^−6^	5.59 × 10^−10^	8.90 × 10^−6^
Zn	4.66 × 10^−4^	4.38 × 10^−8^	2.33 × 10^−5^	3.84 × 10^−4^	3.61 × 10^−8^	1.92 × 10^−5^
Pb	8.87 × 10^−5^	8.35 × 10^−9^	4.44 × 10^−6^	1.46 × 10^−4^	1.37 × 10^−8^	7.28 × 10^−6^
Cu	9.44 × 10^−5^	8.89 × 10^−9^	4.72 × 10^−6^	5.58 × 10^−5^	5.25 × 10^−9^	2.79 × 10^−6^
Cd	6.40 × 10^−7^	6.02 × 10^−11^	3.20 × 10^−8^	4.50 × 10^−7^	4.24 × 10^−11^	2.25 × 10^−8^
Cr	1.27 × 10^−4^	1.19 × 10^−8^	6.35 × 10^−6^	1.16 × 10^−4^	1.09 × 10^−8^	5.81 × 10^−6^
Ni	4.40 × 10^−5^	4.14 × 10^−9^	2.20 × 10^−6^	4.07 × 10^−5^	3.83 × 10^−9^	2.03 × 10^−6^
Mn	6.34 × 10^−4^	5.96 × 10^−8^	3.17 × 10^−5^	7.59 × 10^−4^	7.14 × 10^−8^	3.79 × 10^−5^
(**b**)						
As	3.83 × 10^−5^	1.21 × 10^−9^	6.60 × 10^−6^	3.13 × 10^−5^	9.91 × 10^−10^	5.40 × 10^−6^
Zn	2.46 × 10^−3^	7.78 × 10^−8^	1.41 × 10^−5^	2.03 × 10^−3^	6.41 × 10^−8^	1.17 × 10^−5^
Pb	4.68 × 10^−4^	1.48 × 10^−8^	2.69 × 10^−6^	7.69 × 10^−4^	2.43 × 10^−8^	4.42 × 10^−6^
Cu	4.98 × 10^−4^	1.58 × 10^−8^	2.87 × 10^−6^	2.94 × 10^−4^	9.31 × 10^−9^	1.69 × 10^−6^
Cd	3.38 × 10^−6^	1.07 × 10^−10^	1.94 × 10^−8^	2.38 × 10^−6^	7.51 × 10^−11^	1.37 × 10^−8^
Cr	6.70 × 10^−4^	2.12 × 10^−8^	3.85 × 10^−6^	6.14 × 10^−4^	1.94 × 10^−8^	3.53 × 10^−6^
Ni	3.32 × 10^−4^	7.34 × 10^−9^	1.34 × 10^−6^	2.15 × 10^−4^	6.79 × 10^−9^	1.23 × 10^−6^
Mn	3.34 × 10^−3^	1.06 × 10^−7^	1.92 × 10^−5^	4.01 × 10^−3^	1.27 × 10^−7^	2.30 × 10^−5^

**Table 11:** (**a**) Non-carcinogenic risk values for potentially toxic elements in dust (adults). (**b**) Non-carcinogenic risk values for potentially toxic elements in dust (children).

Potentially Toxic Element	Bus Stop				Parking Lot			
	HQ_ing_	HQ_inh_	HQ_der_	HI	HQ_ing_	HQ_inh_	HQ_der_	HI
(**a**)								
As	2.29 × 10^−2^	5.55 × 10^−6^	3.63 × 10^−2^	5.92 × 10^−2^	1.98 × 10^−2^	4.54 × 10^−6^	2.97 × 10^−2^	4.95 × 10^−2^
Zn	1.55 × 10^−3^	1.46 × 10^−7^	3.88 × 10^−4^	1.94 × 10^−3^	1.28 × 10^−3^	1.20 × 10^−7^	3.20 × 10^−4^	1.60 × 10^−3^
Pb	2.53 × 10^−2^	2.37 × 10^−6^	8.45 × 10^−3^	3.38 × 10^−2^	4.16 × 10^−2^	3.90 × 10^−6^	1.39 × 10^−2^	5.55 × 10^−2^
Cu	2.36 × 10^−3^	2.21 × 10^−7^	3.93 × 10^−4^	2.75 × 10^−3^	1.39 × 10^−3^	1.31 × 10^−7^	2.32 × 10^−4^	1.63 × 10^−3^
Cd	6.40 × 10^−4^	6.02 × 10^−8^	3.20 × 10^−3^	3.84 × 10^−4^	4.50 × 10^−4^	4.24 × 10^−8^	2.25 × 10^−3^	2.70 × 10^−3^
Cr	4.23 × 10^−2^	4.18 × 10^−4^	1.06 × 10^−1^	1.49 × 10^−2^	3.87 × 10^−2^	3.83 × 10^−4^	9.69 × 10^−2^	1.36 × 10^−1^
Ni	2.20 × 10^−3^	2.01 × 10^−7^	4.07 × 10^−4^	2.61 × 10^−3^	2.03 × 10^−3^	1.86 × 10^−7^	3.77 × 10^−4^	2.41 × 10^−3^
Mn	1.35 × 10^−2^	4.26 × 10^−3^	1.32 × 10^−2^	3.09 × 10^−2^	1.61 × 10^−2^	5.10 × 10^−3^	1.58 × 10^−2^	3.70 × 10^−2^
HI	1.11 × 10^−1^	4.69 × 10^−3^	1.68 × 10^−1^	2.84 × 10^−1^	1.21 × 10^−1^	5.49 × 10^−3^	1.59 × 10^−1^	2.86 × 10^−1^
(**b**)								
As	1.28 × 10^−1^	9.84 × 10^−6^	2.20 × 10^−2^	1.50 × 10^−1^	1.28 × 10^−1^	5.55 × 10^−6^	3.63 × 10^−2^	1.64 × 10^−1^
Zn	8.20 × 10^−3^	2.59 × 10^−7^	2.36 × 10^−4^	8.43 × 10^−3^	8.20 × 10^−3^	1.46 × 10^−7^	3.88 × 10^−4^	8.59 × 10^−3^
Pb	1.34 × 10^−1^	4.21 × 10^−6^	5.13 × 10^−3^	1.39 × 10^−1^	1.34 × 10^−1^	2.37 × 10^−6^	8.45 × 10^−3^	1.42 × 10^−1^
Cu	1.25 × 10^−2^	3.92 × 10^−7^	2.39 × 10^−4^	1.27 × 10^−2^	1.25 × 10^−2^	2.21 × 10^−7^	3.93 × 10^−4^	1.29 × 10^−2^
Cd	3.38 × 10^−3^	1.07 × 10^−7^	1.94 × 10^−3^	5.32 × 10^−3^	3.38 × 10^−3^	6.02 × 10^−8^	3.20 × 10^−3^	6.58 × 10^−3^
Cr	2.23 × 10^−1^	7.41 × 10^−4^	6.42 × 10^−2^	2.88 × 10^−1^	2.23 × 10^−1^	4.18 × 10^−4^	1.06 × 10^−1^	3.30 × 10^−1^
Ni	1.16 × 10^−2^	3.56 × 10^−7^	2.47 × 10^−4^	1.19 × 10^−2^	1.16 × 10^−2^	2.01 × 10^−7^	4.07 × 10^−4^	1.20 × 10^−2^
Mn	7.12 × 10^−2^	7.55 × 10^−3^	8.01 × 10^−3^	8.67 × 10^−2^	7.12 × 10^−2^	4.26 × 10^−3^	1.32 × 10^−2^	8.86 × 10^−2^
HI	5.92 × 10^−1^	8.31 × 10^−3^	1.02 × 10^−1^	7.02 × 10^−1^	5.92 × 10^−1^	4.69 × 10^−3^	1.68 × 10^−1^	7.64 × 10^−1^

**Table 12:** Carcinogenic risk values of potentially toxic elements in dust.

	R_inh_								CR	
	Adult				Child				Adult	Child
	As	Cd	Cr	Ni	As	Cd	Cr	Ni		
Bus Stop	1.01 × 10^−12^	9.47 × 10^−8^	1.20 × 10^−7^	2.56 × 10^−9^	1.04 × 10^−10^	6.67 × 10^−9^	1.27 × 10^−9^	1.35 × 10^−9^	2.17 × 10^−7^	9.64 × 10^−8^
Parking Lot	8.24 × 10^−13^	7.80 × 10^−8^	1.97 × 10^−7^	1.51 × 10^−9^	1.46 × 10^−12^	1.38 × 10^−7^	3.5 × 10^−7^	9.28 × 10^−10^	2.77 × 10^−7^	4.89 × 10^−7^

## Data Availability

The original contributions presented in this study are included in this article. Further inquiries can be directed toward the corresponding author.

## Supplementary Materials

The following supporting information can be downloaded at: https://www.mdpi.com/article/10.3390/toxics14070593/s1, Table S1. Spike recovery rates of each element (Mean ± SD); Figure S1. Q-type Hierarchical cluster analysis (HCA) dendrogram for bus stop and parking lot surface dust samples using Ward’s lingkage and Euclidean distance; Figure S2. Principal component analysis(PCA) biplot showing the relationships between bus stop and parking lot amomg potentially toxic elements.

## Abbreviations

The following abbreviations are used in this manuscript:

PTE	potentially toxic element
*I_geo_*	geo-accumulation index
$E_{r}^{i}$	ecological risk index
HI	the hazard index
CR	carcinogenic risk values
ICP-OES	inductively coupled plasma–optical emission spectrometry
GDP	gross domestic product
QA	quality assurance
QC	quality control
