# Supplementary material for: Potentially Toxic Element Contamination of Dust from Bus Stops and Parking Lots in a Developing City, East China: Levels, Spatial Distribution, Source Analysis and Risk Evaluation
Source: Toxics. 2026 Jul 6;14(7):593. doi: 10.3390/toxics14070593 (PMC13417220; doi:10.3390/toxics14070593)
Supplement: Supplementary file 1 [file toxics-14-00593-s001.zip › toxics-4342443-supplementary.pdf]

Supplementary Materials:

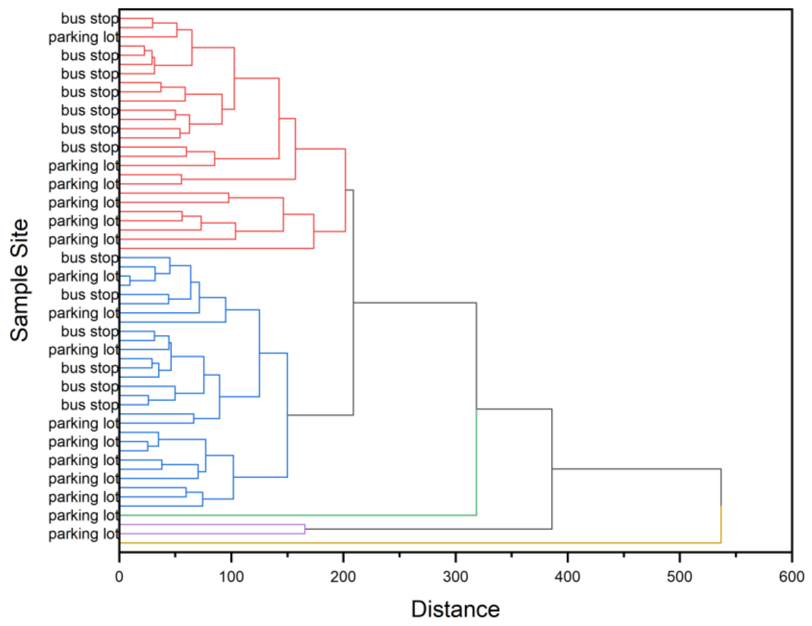

Figure S1. Q-type Hierarchical cluster analysis (HCA) dendrogram for bus stop and parking lot surface dust samples using Ward's lingkage and Euclidean distance.

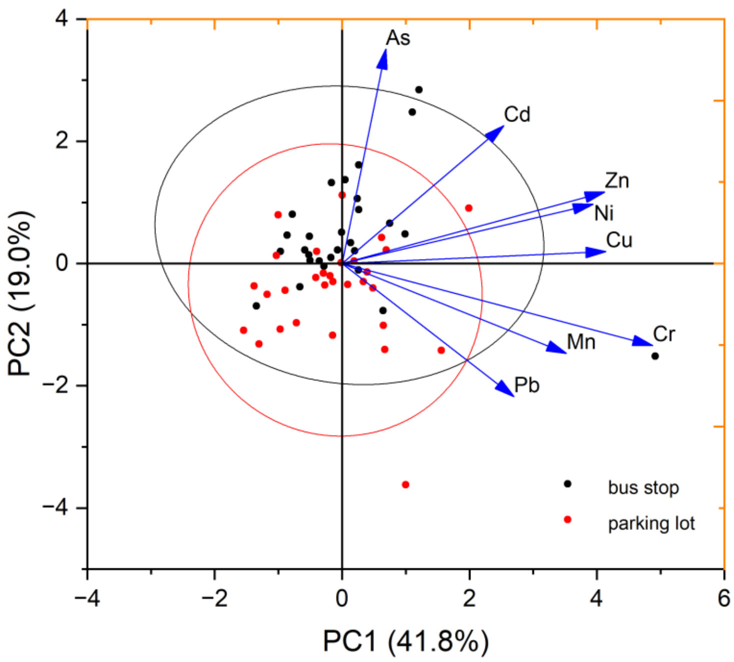

Figure S2. Principal component analysis(PCA) biplot showing the relationships between bus stop and parking lot amomg potentially toxic elements.

Table S1. Spike recovery rates of each element (Mean ± SD)

As	Zn	Cd	Pb	Cu	Ni	Mn	Cr
（83.82±5.40）%	（90.45±5.40）%	（94.23±2.30）%	（84.62±5.08）%	（88.70±2.86）%	（76.66±2.81）%	（79.77±2.16）%	（77.50±3.69）%

The spike recovery rates of all target elements ranged from 76.66% to 94.23%, which fell within the standard acceptable range (70%–120%) for PTE analysis in environmental soil and dust samples. The results demonstrated that the sample pretreatment and instrumental measurement procedures adopted in this study were stable, accurate and reliable.
